# In Search for Reliable Markers of Glioma-Induced Polarization of Microglia

**DOI:** 10.3389/fimmu.2018.01329

**Published:** 2018-06-15

**Authors:** Kacper A. Walentynowicz, Natalia Ochocka, Maria Pasierbinska, Kamil Wojnicki, Karolina Stepniak, Jakub Mieczkowski, Iwona A. Ciechomska, Bozena Kaminska

**Affiliations:** ^1^Laboratory of Molecular Neurobiology, Neurobiology Center, Nencki Institute of Experimental Biology, Polish Academy of Sciences, Warsaw, Poland; ^2^Glia Sp. z o.o, Warsaw, Poland

**Keywords:** microglia, glioma, functional phenotype, transcriptomics, glioma-associated microglia/macrophages

## Abstract

Immune cells accumulating in the microenvironment of malignant tumors are tumor educated and contribute to its growth, progression, and evasion of antitumor immune responses. Glioblastoma (GBM), the common and most malignant primary brain tumor in adults, shows considerable accumulation of resident microglia and peripheral macrophages, and their polarization into tumor-supporting cells. There are controversies regarding a functional phenotype of glioma-associated microglia/macrophages (GAMs) due to a lack of consistent markers. Previous categorization of GAM polarization toward the M2 phenotype has been found inaccurate because of oversimplification of highly complex and heterogeneous responses. In this study, we characterized functional responses and gene expression in mouse and human microglial cultures exposed to fresh conditioned media [glioma-conditioned medium (GCM)] from human U87 and LN18 glioma cells. Functional analyses revealed mutual communication reflected by strong stimulation of glioma invasion by microglial cells and increased microglial phagocytosis after GCM treatment. To define transcriptomic markers of GCM-activated microglia, we performed selected and global gene expression analyses of stimulated microglial cells. We found activated pathways associated with immune evasion and TGF signaling. We performed computational comparison of the expression patterns of GAMs from human GBMs and rodent experimental gliomas to select genes consistently changed in different datasets. The analyses of marker genes in GAMs from different experimental models and clinical samples revealed only a small set of common genes, which reflects variegated responses in clinical and experimental settings. *Tgm2* and *Gpnmb* were the only two genes common in the analyzed data sets. We discuss potential sources of the observed differences and stress a great need for definitive elucidation of a functional state of GAMs.

## Introduction

Tumor microenvironment consists of various non-neoplastic cells that play an important role in tumor growth, progression, and immune response evasion. In glioblastoma (GBM), one of the most aggressive and prevalent primary brain malignancy in adults, tumor-infiltrating microglia and peripheral macrophages are major immune cell population within the tumor. Glioma-associated microglia/macrophages (GAMs) account for up to 30% of tumor mass in human GBMs (1–3) and in experimental glioma models (4–7). Glioma cells secrete various cytokines and chemokines acting as chemoattractants and polarizing factors on the resident microglia [for reviews, see Ref. (8, 9)]. Their role in glioma progression and immunosuppression has been shown in different experimental glioma models (10, 11). Pharmacological or genetic ablation of GAMs reduced tumor growth in mice experimental models of glioma (10, 12, 13). While controversy remains on the magnitude of peripheral macrophage recruitment to gliomas at different steps of tumor progression, there is growing evidence that tumor-specific education of GAMs is expected to determine their effector functions during tumor progression.

In tumor microenvironment, infiltrating macrophages adopt different activation states between antitumor M1 and protumor M2 phenotypes, and these functional phenotypes are defined by differential expression of surface markers, secreted cytokines, and roles in immunoregulation (14). M2 macrophages express frequently genes involved in tissue repair and resolution and have immunosuppressive and immunoregulatory properties. Clinical and mouse model data correlate the accumulation of macrophages with protumor activities. To define a functional phenotype of GAMs *in vivo*, these cells (usually defined as CD11b^+^ cells) have been isolated using immunomagnetic beads or immunosorting and subjected to global gene expression profiling (2, 3, 5, 6). Most of those studies used microarray gene expression profiling, whereas RNA-seq was used to profile CD11b^+^ in human samples. The resulting definitions of transcriptomic-based functional phenotypes of GAMs from human and experimental rodent gliomas are conflicting and indicate either a mixture of M1/M2 phenotypes (3, 5), the M0 phenotype (2), or the M2 phenotype (6). Categorization of GAM polarization using M1/M2 markers reported for peripheral macrophages (14) has been found inaccurate to describe discrete states due to oversimplification of highly complex and heterogeneous responses. There is no consensus and reliable gene expression-based markers.

We have previously characterized rat primary microglia cultures responses to rat C6 glioma-conditioned medium (GCM) and found that factors secreted by glioma cells induce the proinvasive, immunosuppressive polarization of microglia (15, 16). Selected genes were upregulated in cultured microglia, and their expression was also increased in GAMs isolated as CD11b^+^ from C6 gliomas in rats (6). While animal models are a better tool to recreate complex cell–cell interactions between a tumor and its microenvironment, mechanistic analyses of events underlying activation are feasible in cell co-cultures. In this study, we characterized interactions between human glioma and microglial cells and defined gene expression profiles in search for markers of microglia polarization. In addition, we re-evaluated published datasets consisting of differentially expressed genes in GAMs from rat and mouse gliomas, and human GBM samples to identify overlapping or common genes. As a little commonality has been found, we discuss potential sources of variability and prospects of finding reliable markers.

## Materials and Methods

### Microglial Cultures

Primary microglial cultures were prepared from cerebral cortices of P0–P2 old C57BL/6J mice. Following stripping off the meninges and enzymatic brain dissociation the cells were collected and seeded onto the culture flasks. After 48 h, cell cultures were washed three times with phosphate-buffered saline (PBS) to remove debris. Primary cultures were kept in Dulbecco’s modified Eagle’s medium (DMEM) supplemented with 10% fetal bovine serum (FBS), 2 mM l-glutamine, 100 U/mL penicillin, and 100 µg/mL streptomycin (Gibco, MD, USA). Following establishment of glial culture monolayer, microglial cells were isolated by gentle shaking for 1 h at 100 RPM at 37°C. Microglial cells were then collected by centrifugation, counted, checked for viability, and seeded at density 60 × 10^4^ onto 60-mm plastic dishes (for non-adherent cultures). Microglial cultures were used for experiments 48 h after seeding.

Human immortalized SV40 microglia (Applied Biological Materials Inc.) were cultured in Prigrow III Medium (Applied Biological Materials Inc.) supplemented with 10% fetal bovine serum (Gibco, MD, USA) and antibiotics (100 U/mL penicillin, 100 µg/mL streptomycin) in a humidified atmosphere of CO_2_/air at 37°C. Mouse microglial BV2 cells were cultured in DMEM with GlutaMAX™ (DMEM GlutaMAX™) supplemented with 2% fetal bovine serum (Gibco, MD, USA) and antibiotics (100 U/mL penicillin, 100 µg/mL streptomycin) in a humidified atmosphere of CO_2_/air (5%/95%) at 37°C (Heraeus, Hanau, Germany).

### Glioma Cell Cultures

Human GBM cell lines LN18, U87-MG (ATCC, Manassas, VA, USA) were cultured in DMEM supplemented with 10% fetal bovine serum (Gibco, MD, USA) and antibiotics (100 U/mL penicillin, 100 µg/mL streptomycin) in a humidified atmosphere of CO_2_/air (5%/95%) at 37°C (Heraeus, Hanau, Germany).

Patient-derived glioma cell cultures WG4 (GBM WHO grade IV) and IPIN20160420 (GBM WHO grade IV) were developed as previously described (17). Cells were cultured in DMEM/Nutrient Mixture F-12, GlutaMAX™ medium (DMEM/F-12, GlutaMAX™) supplemented with 10% fetal bovine serum (FBS, Gibco, MD, USA) and antibiotics (100 U/mL penicillin, 100 µg/mL streptomycin) in a humidified atmosphere of CO_2_/air (5%/95%) at 37°C (Heraeus, Hanau, Germany).

### GCM and Microglia Treatment

Human glioma cells were seeded onto 60-mm dish at density 75 × 10^4^ in DMEM or DMEM/F12, GlutaMAX™ supplemented with 10% fetal calf serum, and 100 U/mL penicillin and 100 µg/mL streptomycin. Following 24 h, the medium was changed to a microglia culture medium (DMEM or Prigrow III Medium 10% FBS with antibiotics), and glioma cells were cultured for another 24 h. Fresh GCM was collected and used to treat microglial cells. Cells exposed to GCM were collected after 3 and 6 h.

### Functional Tests on Microglia

Morphological changes were assessed in microglial cultures using a co-culture system. Microglial cells were seeded onto round glass cover slips at 8 × 10^4^/well in a 24-well plate and incubated for 48 h. Glioma cells were seeded into the Falcon cell culture inserts with 0.4-µm pores (Falcon #353095) at 6 × 10^4^/insert. After 24 h, the inserts were transferred into the plate with microglial cells and co-cultured for 48 h. Microglial cells were fixed with 2% paraformaldehyde in PBS, permeabilized with 0.1% TRITON X-100, and stained for 30 min at room temperature using Rhodamine–Phalloidin (1:1,000 in PBS). Cells were co-stained with DAPI (4′,6-diamidino-2-phenylindole; 0.001 mg/mL, Sigma) to visualize nuclei. Pictures were acquired using fluorescence microscope (Leica DM4000B, 40× objective). Images were analyzed using ImageJ software, and a ratio of Phalloidin stained area versus DAPI was used for a statistical analysis of cell size.

Phagocytic activity of microglia was assessed using flow cytometry. Microglial cells seeded onto 60-mm dishes and following 48 h, cells were treated for 24 h with fresh human GCM or LPS 100 ng/mL. Fluorescent latex beads were added (50 beads/cell; Sigma L3030) 3 h before read out. Cells were gently scrapped in cold PBS, washed twice and fluorescence was determined by FACSCalibur Flow Cytometer (BD Biosciences). In one run, 10,000 events were collected per sample and a mean fluorescence intensity (MFI) was calculated.

### Gene Expression Analysis

RNA was isolated using RNeasy Mini Kit (QIAGEN, USA), followed by quality and quantity assessment using NanoDrop 2000 Spectrophotometer (Thermo Fisher, USA), and samples were stored at −80°C. RT-PCR was performed using SuperScript III Reverse Transcriptase (Invitrogen, USA) on 500 ng of total RNA and stored at −20°C. Gene expression profiling was performed by quantitative real-time PCR using 10 ng of cDNA in duplicates, using FAST SYBR Green PCR MasterMix (Life Technologies, USA) in 10 µL reaction with a listed set of primers (Table S1 in Supplementary Material). Amplified product was normalized to the endogenous expression of *18S*, and represented as delta Ct values.

Gene expression profiling of human SV40 microglia stimulated with GCM from human glioma LN18 and U87-MG cells was performed with GeneQuery™ Human Microglial Sensome qPCR Array (# GQH-MGS, ScienCell). GeneQuery™ qPCR array is a 96-well plate pre-made format; it contains in each well one validated primer set that recognizes and efficiently amplifies a specific target gene’s cDNA. The primer set recognizes all known transcript variants of the target gene and amplifies only one gene. The array contains eight control genes. The annealing temperature in qPCR analysis was 65°C (with 2 mM Mg^2+^ and no DMSO). Gene expression profiling was performed by qPCR using 10 ng of cDNA from assorted samples from three duplicates for each group (untreated, GCM U87-MG, GCM LN18). The expression of selected genes were tested by qPCR with commercial primers for the following genes *P2RY12, SIGLEC1, SELPLG, IFITM5, PTAFR, TNFRSF1B*, and *GPR84*, purchased from ScienCell Research Laboratories (sequences undisclosed). Each primer set provided lyophilized and ready to use.

Global gene expression analysis using Affymetrix MG-430 PM Strip system (Affymetrix, USA) using GCM treated primary microglia for 6 h (*n* = 3). Samples were run on Bioanalyzer RNA 6000 Pico kit (Agilent, USA) to assess RNA integrity and concentration. The protocol was followed according to the manufacture’s manual using 100 ng of total RNA and samples with RIN > 9.5. Hybridization was performed at 45°C for 16 h and fluidics, and imaging was done as described in the GeneAtlas System User’s Guide (P/N 08-0246). All samples were run at the same time. Data were analyzed using R Studio software and Bioconductor package, and data were deposited into GEO Repository (GSE113370).

### Invasion Assay

Microglial cells were plated onto 24-well plates at the density of 4 × 10^4^. After 24 h, the invasion assay was performed using tissue culture inserts (6.5 mm Transwell^®^ with 8.0 µm Pore Polycarbonate Membrane Insert, Corning, NY, USA) coated with the Growth Factor Reduced Matrigel™ Matrix (BD Biosciences, San Diego. CA, USA). 50 µL of the Matrigel™ Matrix (1 mg/mL) diluted in fresh DMEM medium was dried under sterile conditions (37°C) for 5–6 h. The medium was replaced to fresh one 1 h before seeding the glioma cells into inserts (to a medium for microglial cultures). The LN18, U87-MG, WG4, and IPIN20160420 cells were seeded at a density of 2 × 10^4^/insert on matrigel-covered membrane in serum-reduced medium (2% FBS). The cultures were placed in a 37°C incubator with humidified air containing 5% CO_2_. After 18 h, cells were fixed in ice-cold methanol and cell nuclei stained with DAPI (0.001 mg/mL, Sigma). The membranes from Transwell^®^ inserts were cut out, and images were acquired using fluorescence microscope (Leica DM4000B, 10× objective) of the five independent fields (bottom, top, left, right side, and a center). Numbers of migrating cell nuclei were counted using ImageJ software. All experiments were performed at least three times, in duplicates.

### Statistical Analysis

Each experiment was performed at least three times, on independent passages/cultures, at least in duplicates. Statistical analyses were performed using GraphPad Prism v6.01 (GraphPad Software, Inc., San Diego, CA, USA). The data were plotted as mean ± SD. Differences between the means of the treatments were evaluated using one-way analysis of variance (one-way ANOVA) followed by *post hoc* Dunnett’s multiple comparison test or one-sided paired sample *t*-test (*p*-value) for two groups analysis. *p*-Values <0.05 were considered to be statistically significant and significance is marked as *p < 0.05, **p < 0.01, and ***p < 0.001.

### Comparison of Different Gene Expression Data Sets

Mouse (E-MTAB-2660) and rat (E-MTAB-5050) microarray gene expression data were obtained from the Array Express database. Human RNA-seq expression profiles were obtained from GEO database (GSE80338). The raw microarray data were pre-processed using RMA as described (6). Annotation of probe sets was performed with information provided in the Ensembl database. For RNA-seq data, the already computed FPKM values were used. All data analyses were performed in the R statistical environment and relevant Bioconductor software.

## Results

### Functional Analyses of Microglia Responses to Human Glioma Cells

**Figure 1:**
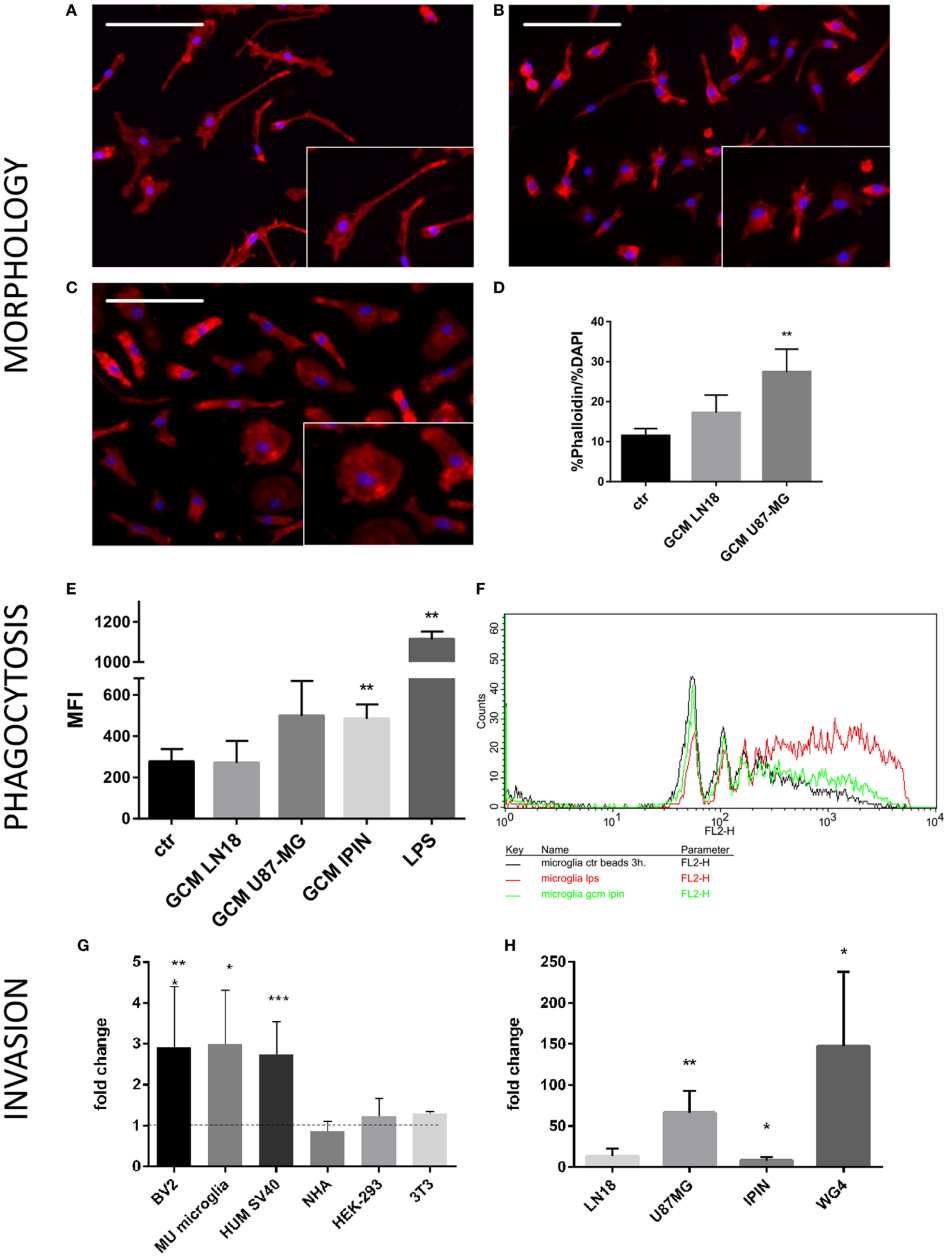
Functional analyses of glioma-induced polarization of murine microglia. Primary murine microglia cultures were co-cultured with human U87-MG or LN18 glioma cells. **(A–C)** Representative images show morphological changes induced in primary murine microglia cultures following co-culture with human U87-MG or LN18 glioma cells. Morphological alterations were visualized by F-actin staining; cell nuclei were co-stained with DAPI. Insets show in higher magnification numerous microglia with amoeboid shape in co-cultures with U87-MG cells. **(D)** Changes were quantified by ratio of percentage of Phalloidin staining to percentage of DAPI staining that is proportional to cell size. **(E,F)** Murine microglia were treated for 24 h with conditioned media [glioma-conditioned medium (GCM)] from human U87-MG, LN18, and IPIN glioma cells or LPS (100 ng/mL), incubated for 3 h with fluorescent beads and subsequently analyzed by flow cytometry. Phagocytosis of fluorescent beads in microglia is represented as mean fluorescence intensity (MFI); graph shows statistically significant groups **(F)** using one-way ANOVA with Dunnett’s multiple comparisons test; *n* ≥ 3. **(G)** Matrigel assay was performed to determine invasion of human U87-MG glioma cells in the presence of different microglial (BV2—immortalized murine microglia, Mu microglia—primary microglial cultures, SV40 immortalized human microglia) or non-microglial cells (NHA—normal human astrocytes, HEP293 cells, and NIH3T3 fibroblasts). Data are calculated as fold change in relation to basal invasion in the absence of microglia. Matrigel invasion data are calculated as means ± SD, *n* ≥ 3 and were analyzed by one-sided paired sample *t*-test; *n* ≥ 3. Differences at *p* < 0.05 were considered significant (***p < 0.001, **p < 0.01, and *p < 0.05). **(H)** Invasiveness of various human glioma cells (IPIN and WG4 are primary patient-derived glioma cultures) is increased to different extent in the presence of BV2 murine microglial cells.

Microglia can respond to various insults or environmental stimuli by changing its morphology, activating phagocytosis, and modulating gene expression (16). Changes from thin/ramified to more amoeboid/round shapes are associated with functional activation *in vitro* and *in vivo* and are useful indicator of activated microglia. We used a co-culture system, in which cells are separated by inserts which allows only the exchange of soluble factors. Murine microglial cells were co-cultured with glioma cells to assess morphological changes induced by conditioned media from U87-MG and LN18 glioma cell cultures (Figures 1A–D). In the presence of glioma cells, we observed profound changes in morphology of microglia and transformation of bipolar cells to the amoeboid shape cells (Figures 1B,C). Insets show representative images of cells with changed morphology in higher magnification. We quantified percentages of amoeboid microglia under various conditions (Figure 1D). U87-MG glioma cells showed the stronger ability than LN18 to induce morphological changes of murine microglia that display more amoeboid/round shapes. Next, we employed flow cytometry to assess phagocytosis of fluorescent beads in primary microglial cultures upon the exposure to GCM from three glioma cultures or LPS, a potent immunomodulator. Phagocytosis was determined as mean fluorescence intensity (MFI) of cells. Graph shows FACS measurements from a representative experiment. There was a significant increase in phagocytosis following the treatment with GCM from primary GBM patient-derived cell cultures (IPIN20160420), and a consistent trend in the increase of phagocytosed beads in microglia treated with U87-MG GCM.

Interactions between microglia and human glioma cells were further investigated by a matrigel invasion assay in a co-culture system. We have previously demonstrated that the presence of rat microglia doubles glioma invasion (18, 19). First, we examined the ability of different microglial cells and other non-microglial cells to increase invasion of U87-MG glioma cells. As shown in the Figure 1G, all tested microglial cells: murine BV2 cells, primary murine microglial cultures, and human SV40 microglial cells significantly increased invasion of U87-MG glioma cells. Non-microglial cells, such as normal human astrocytes, human HEK-293 cells, and murine 3T3 fibroblasts, did not increase invasion of U87-MG glioma cells. As the primary microglial cultures produced most variable results, invasion assay experiments were performed on microglial BV2 cells. As the primary microglial cultures produced most variable results, invasion assay experiments were performed on microglial BV2 cells, and also for comparison on human SV40 cells (Figure S1 in Supplementary Material). We investigated the ability of BV2 microglial cells to support invasion of human U87-MG and LN18 glioma cells, as well as two patient-derived glioma cultures (IPIN20160420 and WG4; Figure 1H). Each glioma cell line had a specific invasion potential under basal conditions, so the increase of invasion in the presence of microglial cells was expressed as a fold change in relation to a cell line control performed for each treatment group. The results show a variable extent of dependence of glioma invasion on microglial cells. Three of four tested glioma cells showed increased in invasion in presence of murine microglial cells. The most prominent increase of invasion was observed in a case of U87-MG and WG4 cells, whereas IPIN20160420 showed moderate increase (Figure 1H). Altogether, our results show that both established and primary cultures of glioma interact in co-culture system, and U87-MG glioma cells exert strong and reproducible effect on murine microglia. Both murine and human microglial cells support invasion of U87 MG glioma cells, while astrocytes and other cells do not have such effect.

### The Expression of Pre-Defined Marker Genes in Murine and Human Microglial Cells Treated With GCM From Human Glioma Cells

**Figure 2:**
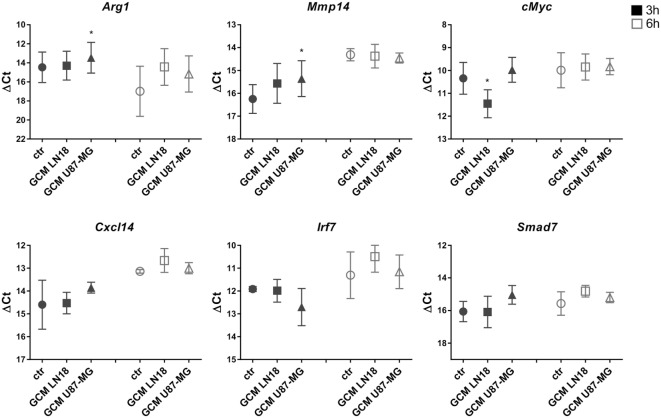
The expression of selected genes in glioma-conditioned medium (GCM) stimulated primary murine microglia cultures. Gene expression was determined by qRT-PCR in microglial cultures left untreated (circles), treated with GCM from LN18 (squares), or U87-MG (triangles) for 3 h (black) and 6 h (white). Data are shown as delta Ct values relative to *18S* expression. Assessment of statistical significance was performed using one-way ANOVA test, followed by Dunnett’s multiple comparison test. The results are calculated as means ± SD, *n* ≥ 3. Differences at *p* < 0.05 were considered as significant (***p < 0.001, **p < 0.01, and *p < 0.05).

We have demonstrated the induction of certain genes in rat microglia cultures exposed for 6 h to GCM from C6 rat glioma cells (15, 16). Therefore, we sought to investigate whether those markers of microglia activation are modulated in murine microglia by GCM from human glioma cells. Primary murine microglia cultures were treated with fresh GCM from LN18 and U87-MG human glioma cells for 3 and 6 h. The expression of *Mmp14, cMyc, Arg1, Ifnb1, Cxcl14, Irf7*, and *Smad7* was assessed with qRT-PCR, and the results are plotted as delta Ct values relative to the endogenous *18S* expression (Figure 2). Increases in the *Mmp14* and *Arg1* expression were detected in microglia exposed to GCM from U87-MG cells for 3 h. Small increases of *Arg1, Cxcl14*, and *Smad7* mRNA levels at 6 h were observed; however, due to large variations between the biological repeats, these changes did not reach significance. None of the tested genes was significantly upregulated by GCM from LN18 glioma cells. The basal expression of selected genes varied between 3 and 6 h. The expression of endogenous *18S* was used as a reference for the amount of cDNA as its expression did not change following treatment.

**Figure 3:**
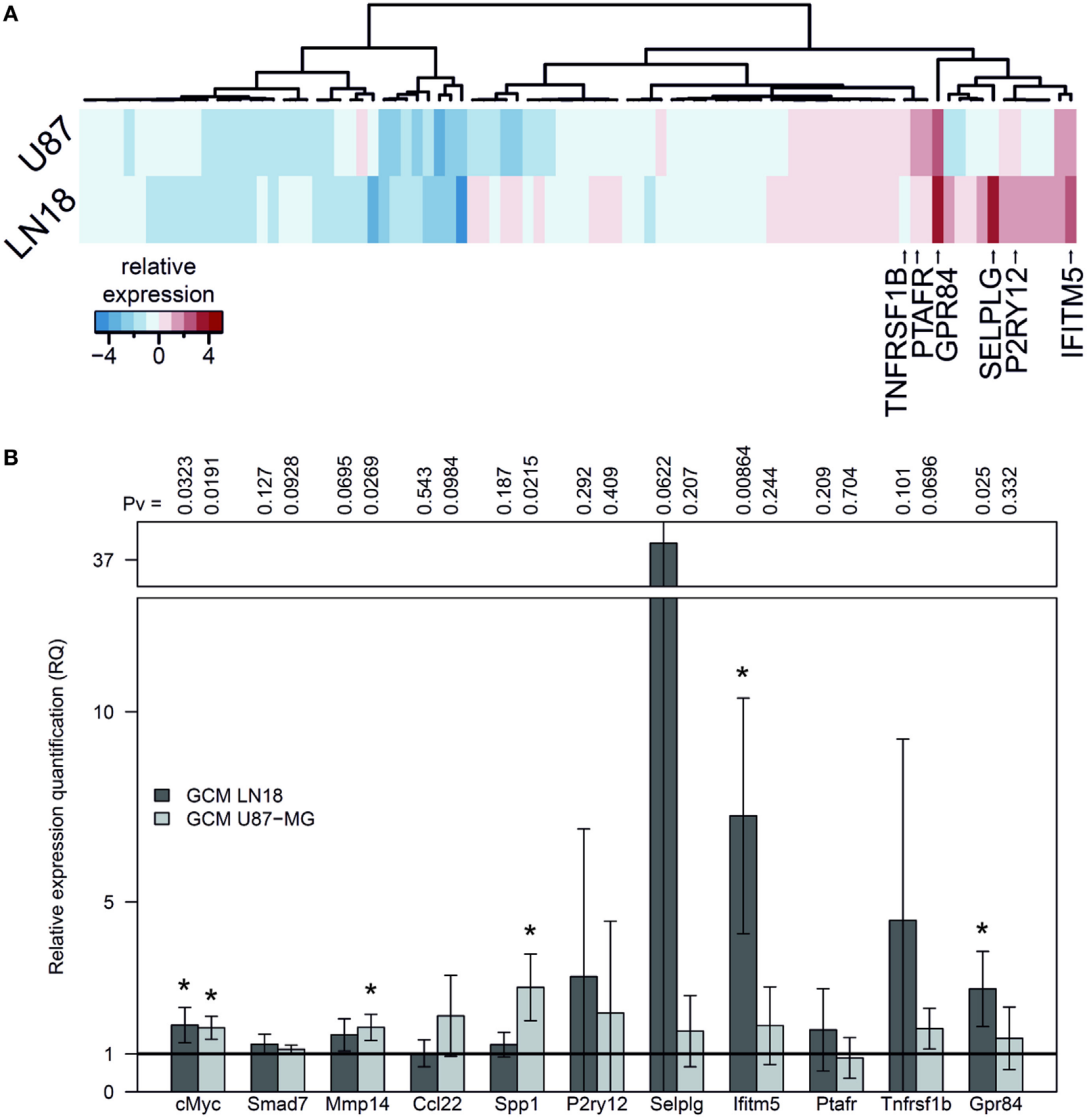
Gene expression in human SV40 microglia polarized by factors secreted by human glioma cells. **(A)** Human Microglial Sensome Gene expression profiling in human SV40 microglia treated by glioma-conditioned medium (GCM) from human LN18 and U87-MG glioma cells was determined with GeneQuery™ Human Microglial Sensome qPCR Array 6 h after GCM addition. The analysis was performed on assorted samples from three replicates for each group (untreated, stimulated with GCM U87-MG or LN18). Heatmap was generated to represent differentially expressed genes and samples. **(B)** The expression of selected genes (*cMYC, SMAD7, MMP14, CCL22*, and *SPP1*) was validated by qPCR in independent cultures of human SV40 microglia treated with GCM from U87-MG or LN18 cells for 6 h. The results are shown as relative quantification (RQ) of GCM stimulated samples compared with untreated controls. Assessment of statistical significance was performed using one-sample *t*-test. The results are calculated as means ± SD, *n* ≥ 3. Differences at *p* < 0.05 were considered as significant (***p < 0.001, **p < 0.01, and *p < 0.05).

We used GeneQuery™ Human Microglial Sensome qPCR Array examining 89 genes to perform gene expression profiling of human SV40 microglial cells polarized by GCM from human LN18 and U87-MG glioma cells. The results, presented as a heatmap, demonstrate moderate similarity in the profiles of upregulated genes in microglia stimulated with GCM U87-MG or LN18. The expression of genes strongly upregulated by either treatment is indicated by arrows and gene names (Figure 3A). The expression of selected, upregulated genes (incorporating *c-MYC, SMAD7*, and *MMP14*) was validated by qPCR in independent cultures of human SV40 microglial cells treated for 6 h with GCM and corroborated the qPCR Array results. A number of genes were significantly upregulated, and we observed the stronger effect after GCM LN18 treatment (Figure 3B).

### Global Gene Expression Analysis in GCM-Treated Mice Microglia

**Figure 4:**
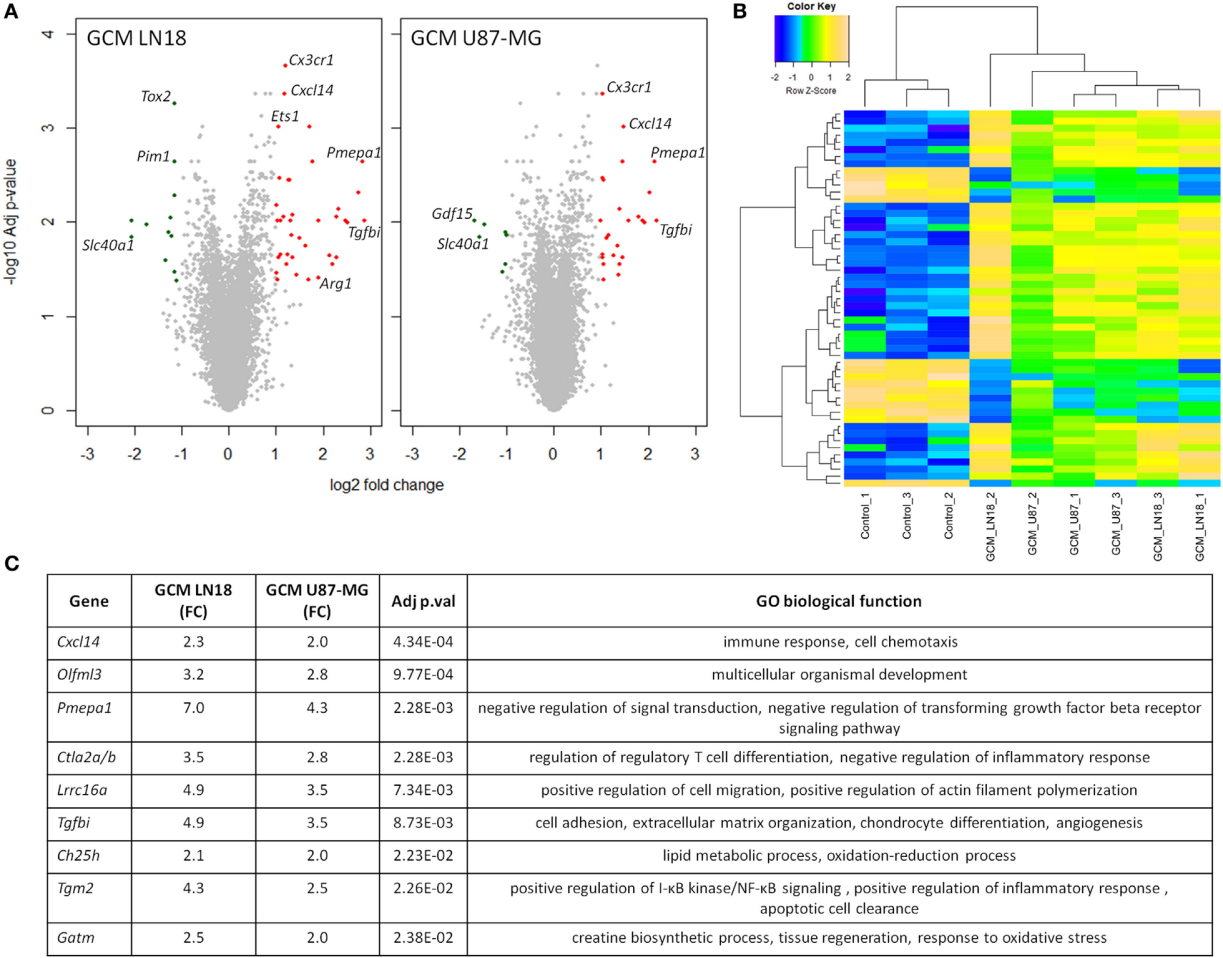
Global gene analysis in primary microglia cultures stimulated with glioma-conditioned medium (GCM) from human glioma cells. Primary microglia cultures were treated for 6 h with GCM. Total RNAs were isolated and subjected to Affymetrix microarray analysis. Data were pre-processed as described in Section “Materials and Methods” and significantly regulated genes are depicted. **(A)** Volcano plots represent log2 fold change versus −log10 adjusted *p*-value in microglia treated with GCM from LN18 and U87-MG glioma cells; upregulated genes are marked in red; downregulated genes in green. **(B)** Heatmap was generated to show significantly differentially expressed genes and sample clustering. **(C)** Selected top significantly upregulated genes in microglia treated with GCM LN18 or U87-MG were listed in a table with fold change expression (FC), adjusted *p*-value, and associated Gene Ontology (GO) biological functions. Significance was assigned to adj *p*-val < 0.05.

As the preselected gene approach did not appear useful in identification of genes modulated during polarization of microglia by human glioma cells, to identify differentially expressed genes, we performed gene expression using Affymetrix arrays. Microarray analysis was performed on murine microglial cells exposed to fresh GCM from LN18 and U87-MG for 6 h. Volcano plots represent differentially expressed genes relative to untreated microglia (Figure 4A). Following the analysis, 36 unique genes were found to be significantly differentially expressed, with 20 genes common in both groups. There were 15 uniquely expressed genes in GCM LN18 and only 1 in GCM U87-MG (*Acss1*) stimulated microglia. Overall, the expression of genes was similar in LN18 and U87 GCM-treated microglia (Figure 4B). We performed validation of selected genes by qPCR and found five of six upregulated by GCM from two cell lines (Figure S2 in Supplementary Material).

Genes upregulated in both groups are associated with immune response, chemotaxis, and signal transduction regulation as defined by Gene Ontology (GO) functions (Figure 4C). Interestingly, we found two upregulated genes that were related to transforming growth factor beta (TGFβ) signaling. Western blot analysis for a phosphorylated Smad2, an active component of TGFβ signaling pathway, was performed on total extracts from microglia polarized with GCM from glioma cells. We found the increased levels of active, phosphorylated Smad2 proteins accumulating at 6 h after GCM treatment (Figure S3 in Supplementary Material).

### *Tgm2* and *Gpnmb* Are Universal Markers of GAMs

Our results show surprisingly low commonality in transcriptomic responses in different models of glioma-microglia interactions. Therefore, we performed reassessment of publicly available datasets for genome-wide analysis of gene expression in GAMs isolated from mouse (5) and rat gliomas (6), and compared the results to human GAMs data sets (2, 3).

**Figure 5:**
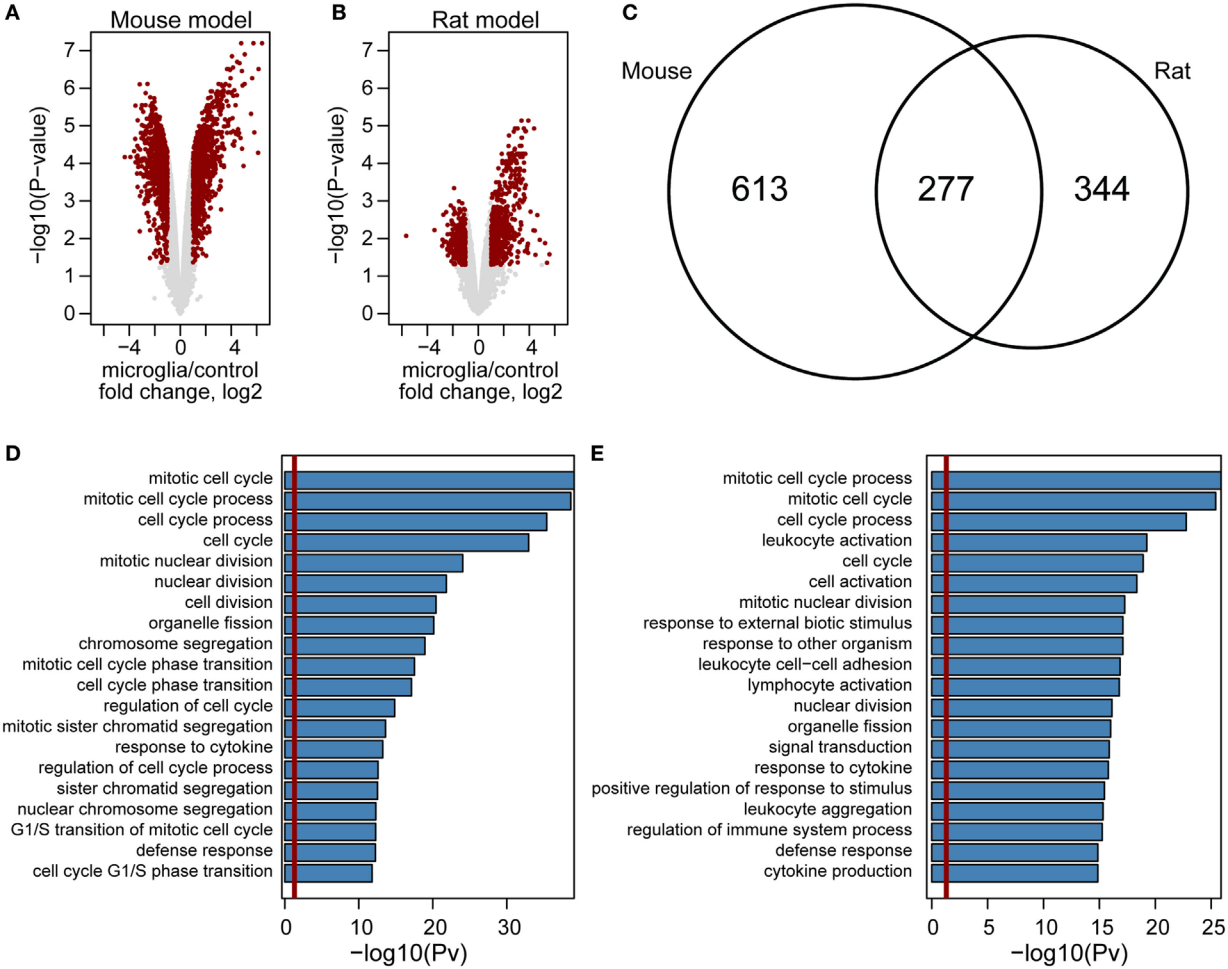
Genome-wide comparison of gene expression in microglia in mouse and rat glioma models. Volcano plot of gene expression changes in glioma-associated microglia/macrophages from mouse **(A)** and rat **(B)** models. The data for all genes are plotted as log2 fold change between microglia and control samples versus the −log10 of the corresponding adjusted *p*-values. Genes selected as significantly different are highlighted as red dots. Venn diagram for overlap of genes upregulated in mouse (left) and rat (right) model **(C)**. Enrichment analysis of Gene Ontology terms within mouse **(D)** and rat **(E)** upregulated genes. Bar plots represent the statistical significance of the enrichment [−log10 (*p*-val)] **(D,E)**.

Although we found hundreds of upregulated genes in either mouse or rat GAMs (Figure 5), the correlation analysis computed using expression changes of orthologs in GAMs versus control microglia did not point to any model which more closely mimics gene expression levels in human GAMs (Figure S4 in Supplementary Material). Nevertheless, we observed that expression changes are more prominent in a mouse model (Figures 5A,B). We identified 890 genes with higher expression in mouse GAMs, when compared with control microglia samples, and 621 of such genes in rat GAMs; 277 of the identified genes were common for both models (Figure 5C). GO analysis revealed that out four of five top enriched GO terms are common for mouse and rat models (Figures 5D,E). Interestingly, GO terms identified only for mouse model were connected mostly with cellular responses, while GO terms identified in a rat model were mainly connected with chromatin and cell cycle. In summary, these results show that despite some similarities, the mouse and rat models represent different pathways of GAM activation.

To determine whether any universal GAM marker might be identified, we set up a comparative analysis of four publically available data sets: (a) MACS sorted CD14^+^ from human GBM versus postmortem tissue and low grade gliomas profiled with microarrays (2); (b) MACS sorted CD11b^+^ from human GBM versus postmortem and epilepsy tissue analyzed by RNA-seq (3); (c) MACS sorted CD11b^+^ from murine GL261 GBM profiled with microarrays (5); (d) FACS sorted CD11b^+^/CD45^low^ from rat C6 gliomas profiled with microarrays (6). Those data were compared with the *in vitro* model presented here—primary murine microglia cultures stimulated with LN18 or U87-MG GCM and profiled with microarrays.

We have found that across all data sets, 129 genes are significantly upregulated (inclusion criteria *p* < 0.05, FC > 2) in at least 2 models, whereas only 4 genes were found to be upregulated in 3 models, and none appeared in 4 or more data sets (Table 1). Downregulated genes (inclusion criteria *p* < 0.05, FC < −2) showed nearly no overlap—only one gene was shared between two data sets. The lack of commonly downregulated genes could be caused by very low number of downregulated genes compared with upregulated across investigated data sets (27 down and 17 up in human CD14^+^; 43 down and 292 up in human CD11b^+^, 78 down and 539 up in murine CD11b^+^; 36 down and 287 up in rat CD11b^+^CD45^low^). Therefore, in further analysis, we focused on the upregulated genes.

**Table 1:** Top markers of glioma infiltrating microglia/macrophages.

	Human CD14^+^		Human CD11b^+^		Murine CD11b^+^		Rat CD11b^+^CD45^low^		Primary murine microglia stimulated with			
									LN18 glioma-conditioned medium (GCM)		U87 GCM	
Gene ID	Gabrusiewicz et al. (2)		Szulzewsky et al. (3)		Szulzewsky et al. (5)		Gieryng et al. (6)		Walentynowicz et al.			
				
	FC	adj. *p*-value	FC	adj. *p*-value	FC	adj. *p*-value	FC	adj. *p*-value	FC	adj. *p*-value	FC	adj. *p*-value

*Tgm2*	–	–	**6.50**	4.60E−02	**6.94**	8.33E−04	**8.12**	9.80E−02*	**4.29**	2.60E−02	**2.46**	2.60E−02
*Gpnmb*	–	–	**8.71**	1.22E−06	**36.02**	2.61E−05	**11.54**	4.16E−02	–	–	**–**	–
*Il2ra/rn*	Upregulated		Upregulated		**4.44**	1.79E−07	**5.24**	2.77E−05						
*Ccr7*	**2.83**	3.05E−02	**3.75**	2.29E−03	–	–	–	–	–	–

*Only genes that were upregulated in the minimum of three models were included*.

*Raw data for Gabrusiewicz et al. (2) were not available, thus the gene expression is indicated based on a heatmap from Figure 6 (2).

Interestingly, all of the four genes overexpressed in three data sets—*Tgm2, Gpnmb, Il2ra*, and *Ccr7* (Table 1), encode proteins that are expressed at the plasma membrane, which makes them good marker candidates. Transglutaminase 2 (Tgm2) was upregulated in human CD11b^+^, murine CD11b^+^, and murine microglia stimulated with LN18 GCM. Rat microglia (CD11b^+^CD45^low^) also expressed *Tgm2* at high level (FC = 8.12), although the upregulation was not significant (*p* = 0.098). Tgm2 is a cross-linking enzyme involved in matrix stabilization, which is necessary for effective phagocytosis of apoptotic cells by macrophages (20). The role of Tgm2 was also shown in tumorigenesis, as it is highly expressed by CD44^high^ (stem cells marker) glioma tissue and Tgm2 inhibition lead to decrease of CD44^high^ cells (21). In turn, upregulation of *Gpnmb* was found for all three investigated species (murine and human CD11b^+^, and rat CD11b^+^CD45^low^). The importance of transmembrane glycoprotein NMB (Gpnmb) was already implied by Szulzewsky et al. (5), who showed its overexpression in mouse and human CD11b^+^ GAMs, and demonstrated an association between increased *Gpnmb* expression and poor patient prognosis.

Interleukins and chemokines are cytokine families frequently overexpressed by GAMs in various glioma models. Nevertheless, we have found that only one member of each family—interleukin 2 receptor alpha (Il2ra) and C-C chemokine receptor type 7 (Ccr7) is overexpressed in three of five analyzed models. Importantly, in contrast to *Tgm2* and *Gpnmb*, overexpression of *Il2ra* and *Ccr7* was found only in human and murine GAMs and did not occur in either a rat experimental model or an *in vitro* model.

### Enriched Functional Groups in GAMs

In addition to screening for a specific GAM marker, we performed a search for enriched GO biological processes. Enriched GO terms that contained genes represented in all data sets are presented in Table 2 (for complete functional analysis results see Table S2 in Supplementary Material). Notably, out of 129 genes upregulated in at least 2 data sets, 68 genes were shared between human CD11b^+^ and rat CD11b^+^CD45^low^ data sets, and only 27 and 22 genes between human CD11b^+^ and murine CD11b^+^, and rat CD11b^+^CD45^low^ and murine CD11b^+^ data sets, respectively. In addition, the most enriched GO term—mitotic cell cycle—contains mainly genes from the human CD11b^+^ and rat CD11b^+^CD45^low^ data sets.

**Table 2:** Co-occurrence of genes from the same functional groups across all data sets.

Data set		Human CD14^+^	Human CD11b^+^	Murine CD11b^+^	Rat CD11b^+^CD45^low^
Genes upregulated in minimum 2 sets/total number of upregulated genes in the set		11/17	96/292	60/539	88/287

Enriched Gene Ontology (GO) biological process	All	Gabrusiewicz et al. (2)	Szulzewsky et al. (3)	Szulzewsky et al. (5)	Gieryng et al. (6)

**Mitotic cell cycle** GO:0000278 adj. *p*-value 9.88 × 10^−9^	*Bub1 Aurkb Bub1 Bub1b Ccnb1 Cdc45 Cdc6 Cdca8 Cdk1 Cdkn3 Cdt1 Cenpf Cep55 Chek1 Cit Clspn Dtl E2f7 E2f8 Espl1 Fanci Foxm1 Gpnmb Gpr132 Kif11 Kif18b Kif23 Kif4 Mad2l1 Mki67 Mybl2 Ncapg Ndc80 Nek2 Nuf2 Nusap1 Pim1 Rad51 Ska1 Ska3 Top2a Tpx2 Ube2c*		*Aurkb Bub1 Bub1b Ccnb1 Cdc45 Cdc6 Cdca8 Cdk1 Cdkn3 Cdt1 Cenpf Cep55 Chek1 Cit Clspn Dtl E2f7 E2f8 Espl1 Fanci Foxm1 Gpnmb Kif11 Kif18b Kif23 Mad2l1 Mki67 Mybl2 Ncapg Ndc80 Nek2 Nuf2 Nusap1 Rad51 Ska1 Ska3 Top2a Tpx2 Ube2c*	*Gpnmb Gpr132 Pim1*	*Aurkb Bub1 Bub1b Ccnb1 Cdc45 Cdc6 Cdca8 Cdk1 Cdkn3 Cdt1 Cenpf Cep55 Chek1 Cit Clspn Dtl E2f7 E2f8 Espl1 Fanci Foxm1 Gpnmb Gpr132 Kif11 Kif18b Kif23 Mad2l1 Mki67 Mybl2 Ncapg Ndc80 Nek2 Nuf2 Nusap1 Pim1 Rad51 Ska1 Ska3 Top2a Tpx2 Ube2c*

**Regulation of cell adhesion**GO:0030155 adj. *p*-value 2.25 × 10^−2^	*Prdm1 Ccr7 Ctla2a Has2 Il10 Il1rn Il2ra Lgals1 Lgals3 Mmp14 Nrp1 Plau Ccl5 Spp1 Tgm2 Tnc Vcam1 Gpnmb*	*Ccl5 Ccr7 Il10 Il1rn Il2ra Spp1*	*Ccr7 Gpnmb Has2 Il2ra Lgals1 Lgals3 Mmp14 Nrp1 Plau Tgm2 Tnc*	*Ccl5 Ccr7 Ctla2a Gpnmb Has2 Il10 Il1rn Il2ra Lgals1 Mmp14 Prdm1 Spp1 Tgm2 Tnc Vcam1*	*Gpnmb Lgals3 Nrp1 Plau Prdm1 Vcam1*

**Cell chemotaxis** GO:0060326 adj. *p*-value 2.50 × 10^−2^	*Ccl5 Ccl18 Ccr7 Cxcl10 Cxcl2 Cxcl9 Jaml Lgals3 Nrp1 Pde4b Spp1 Vcam1*	*Ccl5 Ccl18 Ccr7 Cxcl9 Spp1*	*Ccl18 Ccr7 Cxcl2 Lgals3 Nrp1*	*Ccl5 Ccr7 Cxcl10 Cxcl2 Cxcl9 Pde4b Spp1 Vcam1*	*Cxcl10 Lgals3 Nrp1 Pde4b Vcam1*

**Regulation of response to external stimulus** GO:0032101 adj. *p*-value 2.63 × 10^−2^	*Acp5 Aoah Ccl5 Ccr7 Cd109 Ctla2a Cxcl10 Cxcl2 Cxcl9 Gbp4 Il10 Il2ra Nrp1 Plau Prdm1 Ptgs2 Tgm2 Usp18*	*Ccl5 Ccr7 Cxcl9 Il10 Il2ra Ptgs2*	*Acp5 Ccr7 Cd109 Cxcl2 Il2ra Nrp1 Plau Tgm2*	*Acp5 Ccl5 Ccr7 Cd109 Ctla2a Cxcl10 Cxcl2 Cxcl9 Gbp4 Il10 Il2ra Prdm1 Ptgs2 Tgm2 Usp18*	*Aoah Cxcl10 Gbp4 Nrp1 Plau Prdm1 Usp18*

**Blood vessel morphogenesis** GO:0048514 adj. *p*-value 5.44 × 10^−2^	*Ahr Ccl5 Clic4 Cxcl10 E2f7 E2f8 Foxm1 Has2 Hif1a Lgals3 Nrp1 Plau Prdm1 Prrx1 Ptgs*	*Ccl5 Hif1a Ptgs2*	*Ahr E2f7 E2f8 Foxm1 Has2 Lgals3 Nrp1 Plau Prrx1*	*Ahr Ccl5 Clic4 Cxcl10 Has2 Hif1a Prdm1 Prrx1 Ptgs2*	*Clic4 Cxcl10 E2f7 E2f8 Foxm1 Lgals3 Nrp1 Plau Prdm1*

*Only genes that were upregulated in at least two data sets are included*.

This result is not biased by the size of data sets, since comparable numbers of genes were included for the analysis of human CD11b^+^ and rat CD11b^+^CD45^low^, whereas the murine CD11b^+^ data set was even bigger (Table 2). Therefore, we provide further evidence that the immune infiltrates of human GBMs share more similarities with rat C6 gliomas than with murine GL261 gliomas (6).

Gene Ontology term “Regulation of cell adhesion” consisted of genes which roles in the glioma progression were widely studied: *Il10, Mmp14*, and *Spp1*. Interestingly, upregulation of all of the three genes was found only for the murine CD11b^+^ GAMs, *IL10* and *SPP1* were also found in CD14^+^ human GAMs and *MMP14* in CD11b^+^ human GAMs. In rat, CD11b^+^CD45^low^ *Spp1* was also upregulated; however, the increase did not cross the significance threshold (logFC = 5.39, *p*-adj = 0.074), *Il10* and *Mmp14* were also represented in the rat data set, but no change was noted for those genes (Il10 logFC = 0.98, *p*-adj = 0.536; Mmp14 logFC = 1.27, *p*-adj = 0.03).

Other less known genes (*Lgals1/3, Ctla2a, Il2rn, Il1ra*, and *Prdm1*) play a role in T-cell activity and immune response regulation. Cytotoxic T-lymphocyte-associated protein 2 alpha (Ctl2a) is expressed by activated T-cells; however, its function has not been widely studied (22). Galectins are family of proteins regulating T-cell death, where Galectin 1 (Lgals1) is a strong inducer of T-cell apoptosis, and Galectin 3 (Lgals3) either blocks or induces T-cell death depending whether it occurs intra- or extracellularly (23). *Il1rn* is a gene encoding interleukin 1 receptor antagonist, which functions as an inhibitor of the inflammatory response mediators IL1α and IL1β, whereas *Il2ra* encodes an alpha chain of IL2 receptor and its homodimer form a low-affinity receptor. Positive regulatory domain I-binding factor 1 (Prdm1) in turn, is a transcription factor critical for production of IL-10 cytokine, which expression is restricted to antigen-exposed CD4^+^ and CD8^+^ T-cells (24, 25).

Six genes of chemokine family were found in “Cell chemotaxis” GO category; however, this group appeared to be relatively model specific. Human and murine GAMs (CD11b^+^) shared upregulation of *Ccr7* and *Cxcl2*, murine and rat GAMs shared only *Cxcl10* overexpression. Chemokines unique for each model were human CD14b^+^—*CCL13*; human CD11b^+^—*CCL20*; murine CD11b^+^—*Ccl8, Ccl11, Ccl17, Cxcl16, Cxcl4*; rat CD11b^+^CD45^low^—*Cxcl13*. Among genes of GO terms “Regulation of response to external stimulus” and “Blood vessel morphogenesis,” we found *Ptgs2* encoding prostaglandin endoperoxide synthase 2 (COX-2), an enzyme producing inflammatory prostaglandins. *PTGS2* is overexpressed in many cancers and was shown to play role in enhancing tumor progression and angiogenesis [reviewed in Ref. (34)].

## Discussion

The concept of GAMs playing a key role in GBM pathobiology has been verified in recent studies that demonstrated reduction of glioma growth after microglia ablation or pharmacological inhibition (10, 12, 13). We could use this knowledge to steer GAMs from a tumor supportive and anti-inflammatory phenotype toward an antitumor phenotype. However, translation of such results to a clinic has been found difficult as clinical trials with molecules validated in murine models failed to be successful (26). We still need to separate roles of microglia and macrophages and identify signaling pathways underlying their polarization. Use of CD11bCD45 allows for separation of microglia and bone marrow-derived macrophages that infiltrate the tumor mass in rodent models but this cannot be achieved in human glioma samples, as CD11bCD45 discrimination is challenging in flow cytometry analysis.

We intended to understand mechanisms underlying polarization of microglia driven by factors secreted by human glioma cells in a controlled system. Murine microglia exposed to human glioma undergo morphological alterations, increased phagocytosis, and supported glioma invasion (Figures 1A–H). Several of those responses were mimicked by immortalized BV2 and human SV40 microglial cells. We found that microglia functional responses and gene expression profiles differ when exposed to different human glioma cells. Many previously described markers did not manifest in murine microglia polarized by human glioma cells. It is worth noting that in this study microglial cells were treated for several hours to define early responses and this could be partially responsible for small changes in the pre-defined markers. Nonetheless, activated pathways and biological processes show similarities in microglial responses. One of most established markers of polarized microglia in glioma is Arginase 1 (*Arg1*). *Arg1* reduces levels of l-arginine that is essential for normal T-cell function. The increased Arg1 expression was associated with the immunosuppressive phenotype in microglia/macrophages in rat and mice glioma models (10, 15, 27). A recent study reported that inhibition of *Arg1* with the specific inhibitor in murine gliomas leads to the improved antitumor immune response and reduced tumor size (28). We report the increased *Arg1* expression in murine microglia exposed to human glioma GCM, suggesting the activation of immune-suppressive phenotype in these cells. Interestingly, *Arg1* expression is not significantly upregulated in published data sets of GAMs.

Nonetheless, global gene expression analysis revealed genes associated with immune response and immunosuppression (*Pmepa1, Ctla2a/b, Larc15a, Tgfbi*, and *Tgm2*). Activation of TGFβ signaling pathway has been corroborated by Western blot results and detection of the increased levels of phosphorylated Smad2 in microglia after treatment with GCM from LN18 and U87-MG cultures for 6 h. Signaling associated with TGFβ pathway and increased *Tgfbi* expression have been reported in microglia from GL261 gliomas (5). *Tgm2* expression is also induced by TGFβ pathway, participates in immune responses, and contributes to functions of monocytes/macrophages from migration to differentiation (29).

Interestingly, while GCM from all tested glioma cell lines induced the proinvasive activation of microglia, and in case of LN18 and U87MG increases in gene expression, GCM from U87-MG was particularly potent in induction of phagocytosis, morphological transformation, and tumor invasion support. All those cellular events are regulated in microglia by integrin signaling and mediated by activation of focal adhesion kinase/FAK-PI3K-Akt signaling. We have previously reported that tumor derived osteopontin, a ligand of integrins αvβ3, induces FAK-PI3K-Akt signaling in rat microglia (16). In fact, we found that U87-MG cells express and secrete larger quantities of osteopontin than other glioma cells (29), so it may explain the strongest effects of GCM from U87-MG cells on integrin signaling related events in microglia.

### A Critical Assessment of Gene Expression Markers of Glioma-Induced Microglia Polarization

Classification of macrophage functions according to M1 and M2 phenotypes have been based on *in vitro* studies (30), but those phenotypes failed to be reproduced *in vivo*. It has been suggested that GAMs do not undergo an M2 polarization, but rather present a mixture of M1, and M2a,b,c-specific genes (5).

Our comparative analysis of GAM transcriptomics across different *in vivo* models—human, mouse, and rat, failed to reproduce consistent microglia phenotypes that could be classified according to previously reported gene signatures and showed remarkably low similarity between models. A mouse GL261 model is most commonly used in studies on glioma pathobiology, and thus, it is best characterized. However, our results show a stronger similarity of human GAMs gene expression profiles with transcriptomes of microglia infiltrating rat C6 gliomas, than murine GL261 gliomas (Figure S4 in Supplementary Material; Table 2). Both rat C6 and murine GL261 gliomas are cell line allografts that allow recapitulating the immune system response (31, 32), which might be limited in human xenograft models utilizing immunodeficient animals. C6 cell line produces tumors in outbred Wistars rat and has been reported to mimic best the gene expression changes of human glioma, among other rat cell lines (33). On the other hand, mouse GL261 cells form aggressive tumors in syngeneic C57BL/6 strains and present several molecular alterations that are commonly found in human gliomas: *p53* and *K-ras* mutations, MHCI, and MHCII downregulation (32).

The discrepancies and the low commonality in presented data sets might stem from different sources. The analyzed data sets were collected by different groups, using different methodologies (RNA-seq, microarrays) and different platforms for high-throughput transcriptome analysis, which could lead to an increased rate of false negatives and did not allow us to detect less pronounced effects. Even more important constrain which may hamper our analyses stems from methodological differences in GAM isolation, namely, FACS-based immunosorting versus immunomagnetic beads which may result in different degree of sample purity. Magnetic-activated cell sorting is thought to exert less shear stress on sorted cells than FACS. However, FACS offers a possibility to separate cells based on several markers simultaneously, allows for adjustments in gating strategy, and yields better purity.

Analyzed data sets were collected utilizing distinct markers: CD14 or CD11b or CD11bCD45^low/high^ cells, which may result in different subpopulations with discrete functions. Unification of protocols, better separation of different subpopulations, or single-cell sequencing may bring more conclusive results.

Summarizing, despite of all difficulties we have identified two gene candidates for an universal GAM marker in experimental gliomas—*Tgm2* and *Gpnmb*, which should be further investigated. In addition, our functional analysis shows that pathways regulating the similar cell functions are upregulated across all models. It suggests that studies on universal mechanisms orchestrating an adaptation of the tumor-supporting phenotype by microglia and macrophages should be focused on functional groups rather than on individual genes.

## Ethics Statement

The experiments were performed on glia cultures developed from mice pups. According to EU regulations acquiring tissues from laboratory animals does not require a permission from the Ethical committee.

## Author Contributions

KWalentynowicz and BK design the study, interpreted data, and wrote the manuscript. KWalentynowicz performed most of functional and microarray experiments. NO performed transcriptomic data analyses on public data sets. MP and KWojnicki performed invasion and SqPCR assays. KS performed microarray experiments. JM performed computational analyses. IC developed primary GBM cell cultures. All the authors wrote the manuscript.

## Conflict of Interest Statement

Author MP was employed by company Glia Sp. z o.o. All other authors declare no competing interests.

## Supplementary Material

Figure S1 Matrigel assay was performed to determine invasion of different human glioma cells in the presence of SV40 immortalized human microglia. Data are calculated as fold change in relation to basal invasion in the absence of microglia. Matrigel invasion data are calculated as means ± SD, *n* = 3 and were analyzed by one-sided paired sample *t*-test; *n* ≥ 3. Differences at *p* < 0.05 were considered as significant (****p* < 0.001, ***p* < 0.01, and **p* < 0.05).

Figure S2 Microarray gene expression validation. Five genes were selected for validation of the microarray gene expression analysis (*Tgm2, Cx3cr1, Tgfbi, Tmem37*, and *Socs2*). Expression is presented as delta Ct values in control, glioma-conditioned medium (GCM) LN18, and GCM U87-MG treated for 6 h. Validation was performed on separate experiments *n* = 4; ***p* < 0.001, **p* < 0.05.

Figure S3 Western blot analysis of total extracts from microglia polarized with glioma cells for active components of transforming growth factor beta signaling pathway revealed the increased levels of active, phosphorylated SMAD2 proteins accumulating at 3 and 6 h after glioma-conditioned medium (GCM) treatment. Immunoblots were stripped and re-probed first with an antibody recognizing total SMAD2 followed by detection of actin (used as a loading control).

Figure S4 Correlation of gene expression patterns in various datasets. A heatmap shows Spearman’s correlation coefficients between microglia activation (log2 fold changes) for samples collected from human, mouse, and rat samples.
