# Supplementary figures and images for: In Search for Reliable Markers of Glioma-Induced Polarization of Microglia
Source: Front Immunol. 2018 Jun 15;9:1329. doi: 10.3389/fimmu.2018.01329 (PMC6013650; doi:10.3389/fimmu.2018.01329)

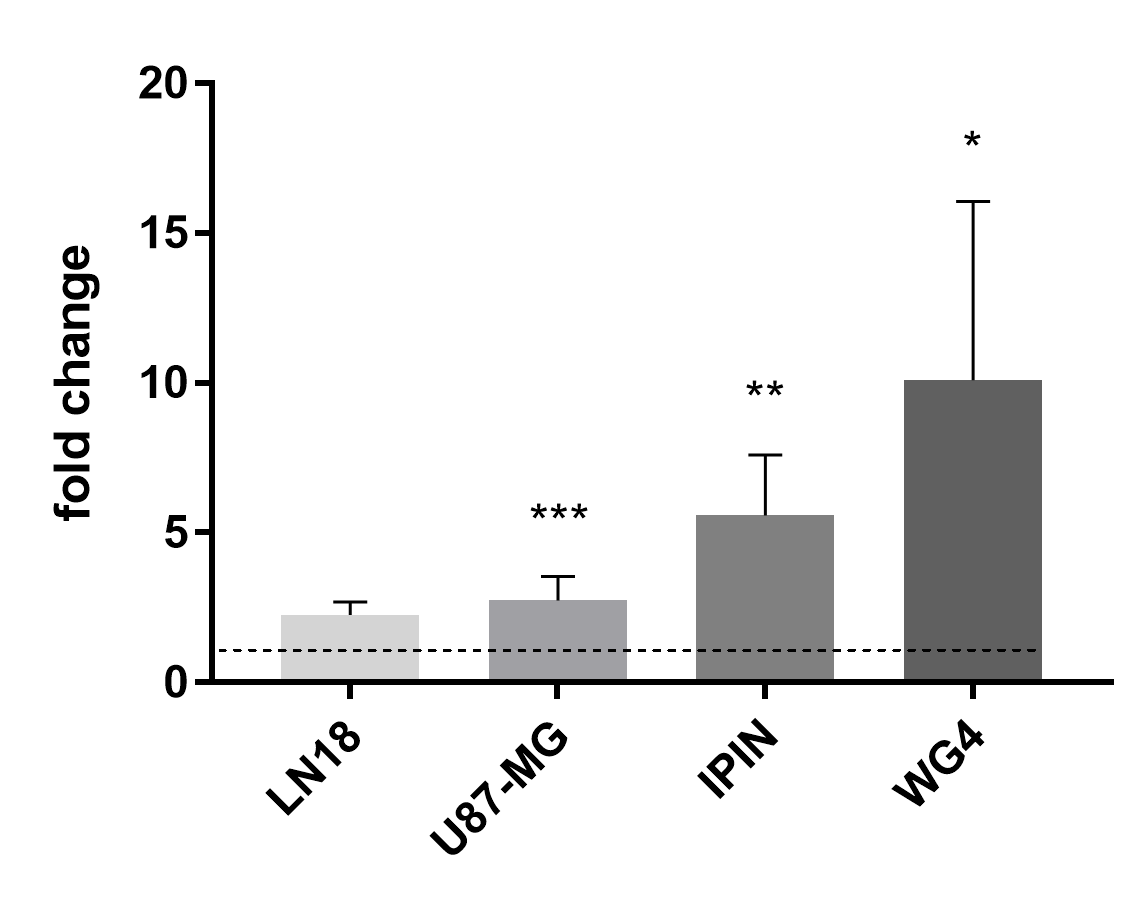

Supplement: Figure S1 — Matrigel assay was performed to determine invasion of different human glioma cells in the presence of SV40 immortalized human microglia. Data are calculated as fold change in relation to basal invasion in the absence of microglia. Matrigel invasion data are calculated as means ± SD, n = 3 and were analyzed by one-sided paired sample t-test; n ≥ 3. Differences at p < 0.05 were considered as significant (***p < 0.001, **p < 0.01, and *p < 0.05). [file Image_1.tif]

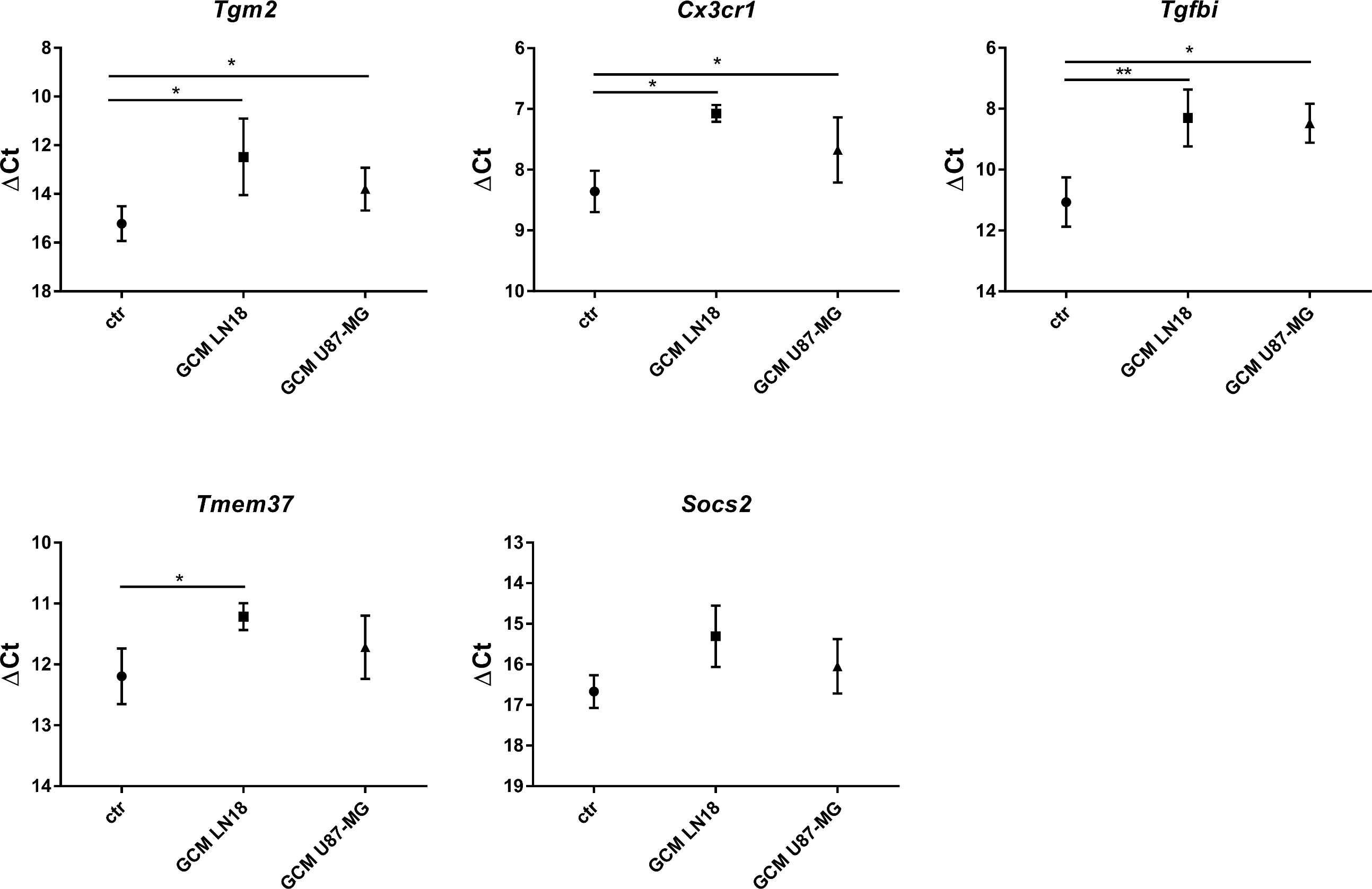

Supplement: Figure S2 — Microarray gene expression validation. Five genes were selected for validation of the microarray gene expression analysis (Tgm2, Cx3cr1, Tgfbi, Tmem37, and Socs2). Expression is presented as delta Ct values in control, glioma-conditioned medium (GCM) LN18, and GCM U87-MG treated for 6 h. Validation was performed on separate experiments n = 4; **p < 0.001, *p < 0.05. [file Image_2.tif]

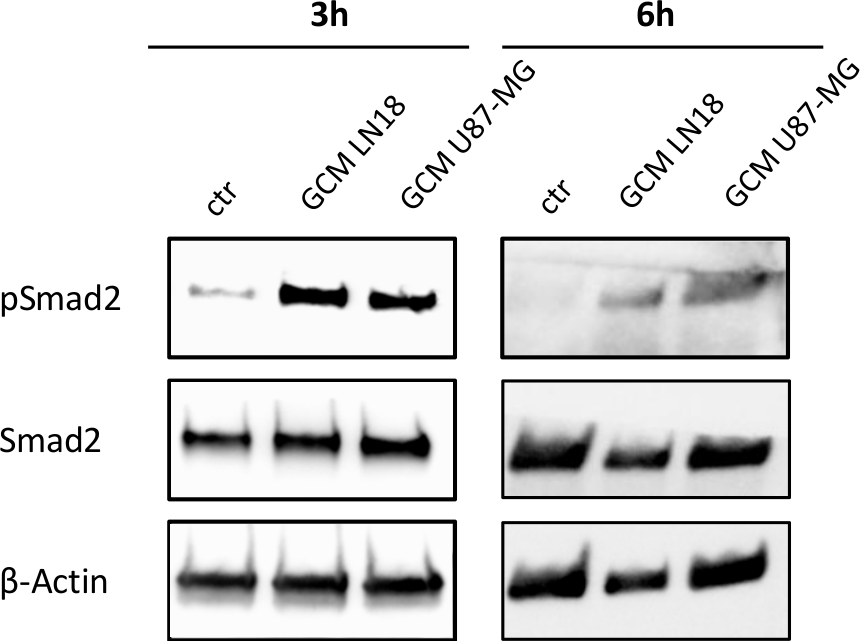

Supplement: Figure S3 — Western blot analysis of total extracts from microglia polarized with glioma cells for active components of transforming growth factor beta signaling pathway revealed the increased levels of active, phosphorylated SMAD2 proteins accumulating at 3 and 6 h after glioma-conditioned medium (GCM) treatment. Immunoblots were stripped and re-probed first with an antibody recognizing total SMAD2 followed by detection of actin (used as a loading control). [file Image_3.tif]

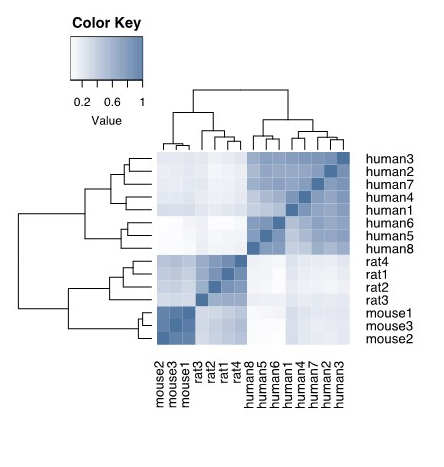

Supplement: Figure S4 — Correlation of gene expression patterns in various datasets. A heatmap shows Spearman’s correlation coefficients between microglia activation (log2 fold changes) for samples collected from human, mouse, and rat samples. [file Image_4.tif]
