# Supplementary material for: In Search for Reliable Markers of Glioma-Induced Polarization of Microglia
Source: Front Immunol. 2018 Jun 15;9:1329. doi: 10.3389/fimmu.2018.01329 (PMC6013650; doi:10.3389/fimmu.2018.01329)
Supplement: Supplementary file 5 [file Data_Sheet_1.docx]

Supplementary Table S1. The list of primers used for qRT-PCR

| Gene | Forward | Reverse |
| --- | --- | --- |
| *18S* | CGGACATCTAAGGGCATCACA | AACGAACGAGACTCTGGCATG |
| *cMYC* | AAAACCAGCAGCCTCCCGCGA | AATACGGCTGCACCGAGTCGT |
| *SMAD7* | TCCTCCGCTGAAACAGGGGGAAC | AGTGTGACCGATCCCCAGGCTC |
| *MMP14* | CTCGGCCCAAAGCAGCAGCT | GCGATGGCCGCTGAGAGTGA |
| *CCL22* | CGTGATTACGTCCGTTACCG | AAGGTTAGCAACACCACGC |
| *SPP1* | AAGCAGCTTTACAACAAATACCCA | TACTTGGAAGGGTCTGTGGGG |
| *Arg1* | TCAAAAGGACAGCCTCGAGGAGGG | AGGTCCCCGTGGTCTCTCACG |
| *Mmp14* | GCAACTTCAGCCCCGAAGCCT | GGAACACCACAGCGAGGGCG |
| *cMyc* | CCCGCGATCAGCTCTCCTGAA | TCACGTTGAGGGGCATCGTCG |
| *Cxcl14* | CAGAGAGAGGCGTGCTTGAA | CAGGAGCAGCAGGAGCAG |
| *Irf7* | GAGTCTGGGGCAGACCCCGT | CAAGGCTGCGCTCGGTGAGA |
| *Smad7* | ACCTTCCTCCGCGGAAACCG | CCACGCACCAGTGTGACCGA |
| *Tgm2* | GACAATGTGGAGGAGGGATCT | CTCTAGGCTGAGACGGTACAG |
| *Cx3cr1* | GAGTATGACGATTCTGCTGAGG | CAGACCGAACGTGAAGACGAG |
| *Tgfbi* | GCGACTTGCCCCTGTCTAT | AACTGAGAGAAACTGGCGGG |
| *Tmem37* | AGTGCGACAGCTAGGCCA | AGCTTGTGGGCCTGCG |
| *Socs2* | AGTTCGCATTCAGACTACCTACT | TGGTACTCAATCCGCAGGTTAG |
