# Supplementary material for: In Search for Reliable Markers of Glioma-Induced Polarization of Microglia
Source: Front Immunol. 2018 Jun 15;9:1329. doi: 10.3389/fimmu.2018.01329 (PMC6013650; doi:10.3389/fimmu.2018.01329)
Supplement: Supplementary file 6 [file Data_Sheet_2.PDF]

			FC>2	p adj >0.05	
all	**Gabrusiewicz**	**Szulzewsky 2016**	**Szulzewsky 2015**	**Gieryng 2017**	**Walentynov**
125	**11 out of 17**	**96 out of 292**	**60 out of 539**	**88 out of 287**	**7 out of 26**
CCR7	CCL18	ACP5	Acp5	Amica1	*AI504432*
GPNMB	CCL5	ADAMDEC1	Adamdec1	Aoah	*Ch25h*
IL2RA	CCR7	AHR	Ahr	Apold1	*Ctla2a*
TGM2	CD163	APLN	AI504432	Aspm	*Gatm*
ACP5	CXCL9	ARRDC4	Amica1	Aurkb	***Socs2***
ADAMDEC1	HIF1A	ASPM	Aoah	Brip1	*Tgfbi*
AHR	IL10	ATP6V0D2	Apln	Bub1	*Tgm2*
Aoah	IL1RN	AURKB	Apold1	Bub1b	
Apln	IL2RA	BRIP1	Arrdc4	Casc5	
Apold1	PTGS2	BUB1	Atp6v0d2	Ccnb1	
Arrdc4	SPP1	BUB1B	Ccl5	Cdc45	
ASPM		CASC5	Ccr7	Cdc6	
ATP6V0D2		CCL18	Cd109	Cdca8	
AURKB		CCNB1	Ch25h	Cdk1	
BRIP1		CCR7	Chl1	Cdkn3	
BUB1		CD109	Clic4	Cdt1	
BUB1B		CD163	Csf2rb	Cenpf	
CASC5		CDC45	Ctla2a	Cenpk	
CCL18		CDC6	Cxcl10	Cenpm	
CCL5		CDCA8	Cxcl2	Cep55	
CCNB1		CDK1	Cxcl9	Chek1	
CD163		CDKN3	Fabp5	Cit	
CDC45		CDT1	Gas2l3	Ckap2l	
CDC6		CENPF	Gatm	Clic4	
CDCA8		CENPK	Gbp4	Clspn	
CDK1		CENPM	Gpnmb	Csf2rb	
CDKN3		CEP55	Gpr132	Cxcl10	
CDT1		CHEK1	Gpx3	Dlgap5	
CENPF		CHL1	Has2	Dtl	
CENPK		CIT	Hif1a	E2f7	
CENPM		CKAP2L	Htr7	E2f8	
CEP55		CLSPN	Ifit1	Esco2	
CHEK1		CXCL2	Igfbp3	Espl1	
CHL1		DLGAP5	Il10	Exo1	
CIT		DTL	Il1rn	Fanci	
CKAP2L		E2F7	Il2ra	Foxm1	
CLSPN		E2F8	Ldlrad3	Gas2l3	
CXCL2		ESCO2	**Lgals1**	Gbp4	
Cxcl9		ESPL1	Lgals3bp	Gpnmb	
Cxcl10		EXO1	Ly9	Gpr132	
DLGAP5		FABP5	Mdfic	Gpx3	
DTL		FANCI	Mmp14	Gtse1	
E2F7		FOXM1	Nceh1	Hjurp	
E2F8		GPNMB	Pde4b	Ifit1	
ESCO2		GTSE1	Pim1	**Kif11**	
ESPL1		HAS2	Plod2	**Kif18b**	
EXO1		HJURP	Ppic	**Kif23**	

FABP5	HTR7	Prdm1	**Kif4a**
Gas2l3	IGFBP3	Prdx1	Kifc1
Gbp4	IL2RA	Prr11	**Lgals3**
Gpx3	**KIF11**	Prrx1	Lgals3bp
Has2	**KIF18B**	Ptgs2	Mad2l1
Ifit1	**KIF23**	Slc7a11	**Mcm10**
Igfbp3	**KIF4A**	Socs2	**Mcm2**
Il10	KIFC1	Spp1	**Mcm4**
Il1rn	LDLRAD3	Tgm2	Melk
**KIF11**	**LGALS1**	Tnc	Mki67
**KIF18B**	**LGALS3**	Tuba4a	Mybl2
**KIF23**	LY9	Usp18	Ncapg
**KIF4A**	MAD2L1	Vcam1	Ndc80
**LGALS1**	**MCM10**		Nek2
**LGALS3**	**MCM2**		Nrp1
**MCM10**	**MCM4**		Nuf2
**MCM2**	MDFIC		Nusap1
**MCM4**	MELK		Pde4b
MDFIC	MKI67		Pim1
MELK	MMP14		Pkmyt1
MKI67	MYBL2		Plau
MMP14	NCAPG		Polq
MYBL2	NCEH1		Prc1
NCAPG	NDC80		Prdm1
NCEH1	NEK2		Prdx1
NDC80	NRP1		Prr11
NEK2	NUF2		Rad51
NRP1	NUSAP1		Rad51ap1
NUF2	PKMYT1		Rai14
NUSAP1	PLAU		Rrm2
Pde4b	PLOD2		Sdc1
Pim1	POLQ		Ska1
Pkmyt1	PPIC		Ska3
Plau	PRC1		Slc7a11
Polq	PRRX1		Top2a
Prc1	RAD51		Tpx2
Prdm1	RAD51AP1		Tuba4a
Prrx1	RAI14		Ube2c
Ptgs2	RRM2		Usp18
Rad51	SDC1		Vcam1
Rrm2	SKA1		Wdhd1
Sdc1	SKA3		
Ska1	TGFBI		
Ska3	TGM2		
Slc7a11	TNC		
Spp1	TOP2A		
TGFBI	TPX2		
TOP2A	UBE2C		
TPX2	WDHD1		

UBE2C
Usp18
Vcam1
Wdhd1
AI504432
Amica1
Cd109
Ch25h
Clic4
Csf2rb
Ctla2a
Gatm
Fanci
Foxm1
Gpr132
Gtse1
HIF1A
HJURP
HTR7
KIFC1
LDLRAD3
Lgals3bp
Ly9
MAD2L1
PLOD2
PPIC
Prdx1
Prr11
Socs2
RAD51AP1
RAI14
TNC
Tuba4a
